# The role of serine metabolism in lung cancer: From oncogenesis to tumor treatment

**DOI:** 10.3389/fgene.2022.1084609

**Published:** 2023-01-09

**Authors:** Xijia Zhou, Chang Tian, Yingshu Cao, Min Zhao, Ke Wang

**Affiliations:** Department of Respiratory and Critical Care Medicine, The Second Hospital of Jilin University, Changchun, China

**Keywords:** serine metabolism, lung cancer, PHGDH, PSAT1, PSPH

## Abstract

Metabolic reprogramming is an important hallmark of malignant tumors. Serine is a non-essential amino acid involved in cell proliferation. Serine metabolism, especially the *de novo* serine synthesis pathway, forms a metabolic network with glycolysis, folate cycle, and one-carbon metabolism, which is essential for rapidly proliferating cells. Owing to the rapid development in metabolomics, abnormal serine metabolism may serve as a biomarker for the early diagnosis and pathological typing of tumors. Targeting serine metabolism also plays an essential role in precision and personalized cancer therapy. This article is a systematic review of *de novo* serine biosynthesis and the link between serine and folate metabolism in tumorigenesis, particularly in lung cancer. In addition, we discuss the potential of serine metabolism to improve tumor treatment.

## Introduction

Emerging evidence suggests that metabolic reprogramming of cancer not only affects tumor progression and molecular pathways but also regulates the tumor immunochemical microenvironment and drug resistance to chemotherapy. Tumor cells satisfy specific bioenergetic and biosynthetic needs through metabolic reprogramming. However, abnormal accumulation of cellular metabolites can stimulate cell carcinogenesis and promote tumor progression (Pavlova and Thompson, 2016; Martínez-Reyes and Chandel, 2021). Metabolic reprogramming involves a series of complex metabolic changes, including increased nutrient intake, enhanced glycolytic metabolism, altered amino acid metabolism, abnormal redox homeostasis, and aberrant fatty acid oxidation (Boroughs and DeBerardinis, 2015). These metabolic changes often occur simultaneously and interact with one another. In particular, serine metabolism is closely related to glycolytic reactions and one-carbon (1C) unit metabolism and provides essential substrates for the biosynthesis of molecules required for cell proliferation, thus playing an important role in tumorigenesis and immunity (Rodriguez et al., 2019; Kurniawan et al., 2020). In this paper, we provide a detailed review of the process of serine metabolism and summarize recent advances in serine metabolism in cancer, especially lung cancer.

### *De novo* serine synthesis pathway

**FIGURE 1:**
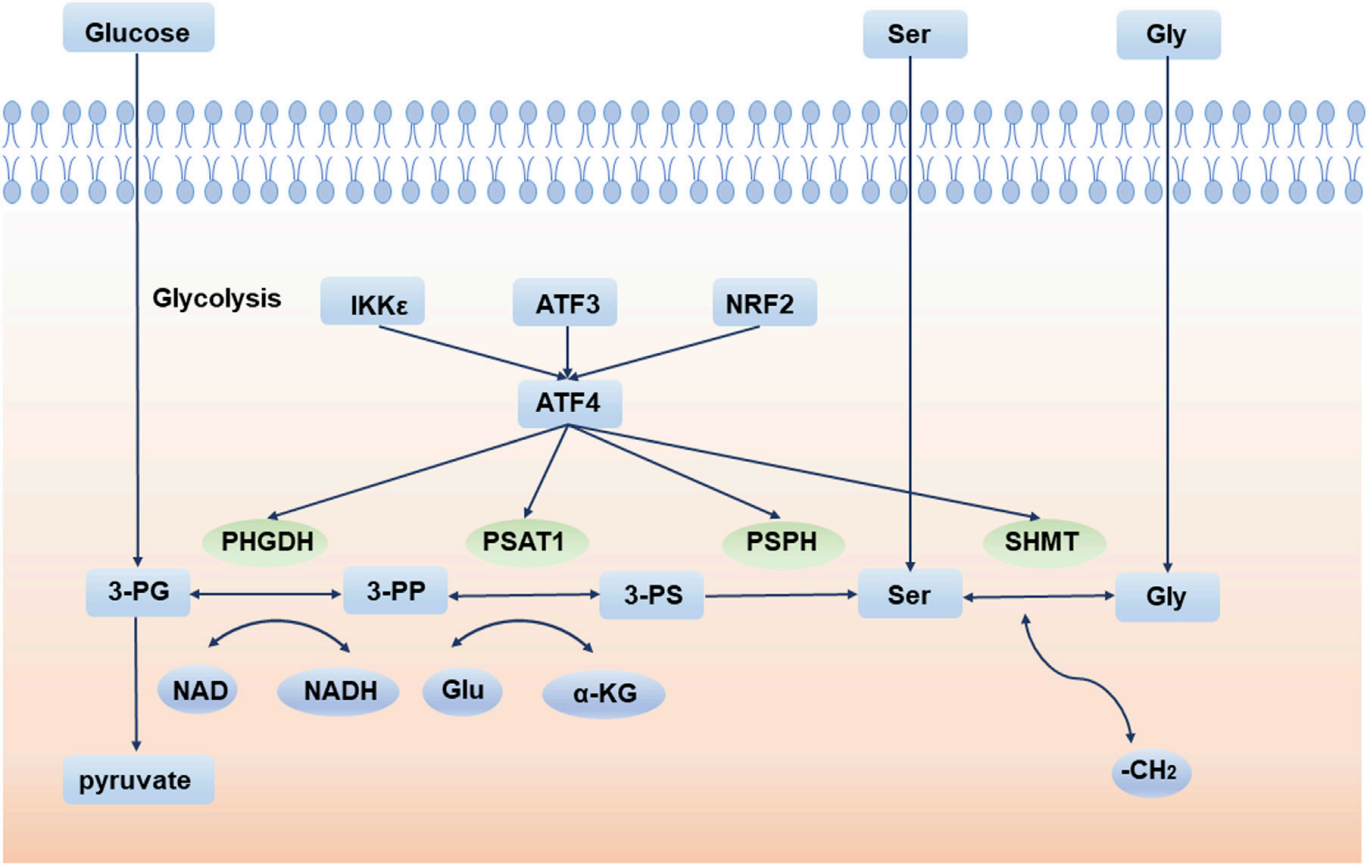
*De novo* serine synthesis pathway and its regulator. 3-PG is converted to serine by PHGDH, PSAT1, and PSPH. Serine is then converted to glycine by SHMT. 3-PG: 3-phosphoglycerate; 3-PP: 3-phosphohydroxypyruvate; 3-PS: 3-phosphoserine; PHGDH: 3-phosphoglycerate dehydrogenase; PSAT1: phosphoserine aminotransferase; PSPH: phosphoserine phosphatase; SHMT: serine hydroxymethyl transferase; Ser: serine; Gly: glycine; Glu: glutamate. IKKε: IκB Kinase ε; ATF3: activating transcription factor 3; NRF2: NFE2 like bZIP transcription factor 2; ATF4: activating transcription factor 4.

Deregulation of cellular energetics is one of the most pervasive hallmarks of cancer (Hanahan and Weinberg, 2011). Compared to normal differentiated cells, cancer cells prefer aerobic glycolysis to provide energy for cell growth. This phenomenon is also called the “Warburg effect” (Vander Heiden et al., 2009). Aerobic glycolysis produces ATP inefficiently but confers many advantages to tumor cells, especially in supporting cell anabolic reactions (Lunt and Vander Heiden, 2011). Therefore, the *de novo* serine synthesis pathway (SSP) is an important turning point for glucose conversion. SSP is the process by which the glycolytic intermediate, 3-phosphoglycerate (3-PG), is converted to serine by 3-phosphoglycerate dehydrogenase (PHGDH), phosphoserine aminotransferase (PSAT1), and phosphoserine phosphatase (PSPH). Serine is then converted to glycine by serine hydroxymethyl transferase (SHMT) (Figure 1).

Glucose enters the serine pathway from the glycolysis intermediate product, 3-PG, which is the origin of SSP. Serine, the product of SSP, is a non-essential amino acid (NEAA) that is an essential precursor for protein, nucleic acid, and lipid synthesis (Amelio et al., 2014). For example, L-serine and palmitoyl CoA are critical sphingolipid units. In astrocytes, the process by which glucose metabolism produces serine and further synthesizes sphingolipids, is essential for brain development and neuronal survival (Hirabayashi and Furuya, 2008). Conversely, toxic deoxysphinganine (doxSA), which is produced upon serine depletion, damages both vascular and nervous systems (Gantner et al., 2019). More importantly, serine metabolism and glycine synthesis are inextricably linked in biology, providing 1C units for the sustainability of nucleotides, S-adenosylmethionine (SAM), reduced nicotinamide adenine dinucleotide phosphate (NADPH), and glutathione (GSH) (Amelio et al., 2014).

Under normal circumstances, serine in cells comes from two sources: food intake and intracellular synthesis from the SSP pathway. When the exogenous serine intake is insufficient, SSP is enhanced to produce more serine. Activating transcription factor 4 (ATF4) is the main regulator of SSP and directly binds to and modulates the transcription of the SSP core enzymes PHGDH, PSAT1, PSPH, and SHMT (Wortel et al., 2017). In response to amino acid starvation or endoplasmic reticulum (ER) stress, ATF4 is activated to initiate the transcription of downstream molecules and adapt to environmental stress (B'Chir et al., 2013). Furthermore, a variety of regulators indirectly regulate the SSP pathway *via* ATF4. For example, experiments have indicated that activating transcription factor 3 (ATF3) is crucial for the activation of the SSP pathway, especially when serine is deprived. ATF4 is first activated during serine deprivation, and then ATF3 is quickly activated depending on ATF4. ATF3 not only increases the expression of ATF4, but also promotes the expression of PHGDH, PSAT1, and PSPH by interacting with their enhancers and promoters. ATF3 also recruits E1A-binding protein p300 to transactivate key SSP enzymes (Li et al., 2021a). Another example is NFE2-like bZIP transcription factor 2 (NRF2), an essential transcription factor frequently deregulated in non-small cell lung cancer (NSCLC), which regulates SSP enzymes by targeting ATF4. Experiments show that after knockdown of NRF2, ATF4 is decreased at the protein level but does not change significantly at the transcription level. As NRF2 does not bind to key SSP enzyme promoters directly, it can be inferred that NRF2 regulates serine biosynthesis by promoting the expression of ATF4 (DeNicola et al., 2015). Additionally, IκB Kinase ε (IKKε), a key kinase that affects tumors and inflammation, is overexpressed in these tumors. IKKε reduces glucose-derived pyruvate utilization in the TCA cycle to decrease mitochondrial function, thus activating ATF4 (Xu et al., 2020). Lysine demethylase 4C (KDM4C) regulates serine pathway genes by removing the trimethylation modification of H3 lysine 9 (H3K9), which requires the involvement of ATF4 (Zhao et al., 2016). The GCN2-ATF4 (Pathria et al., 2018) and eIF2α-ATF4 (B'Chir et al., 2013) signaling pathways also play important roles in the process of tumor cells coping with amino acid starvation and maintaining cell proliferation.

### SSP and one-carbon metabolism

**FIGURE 2:**
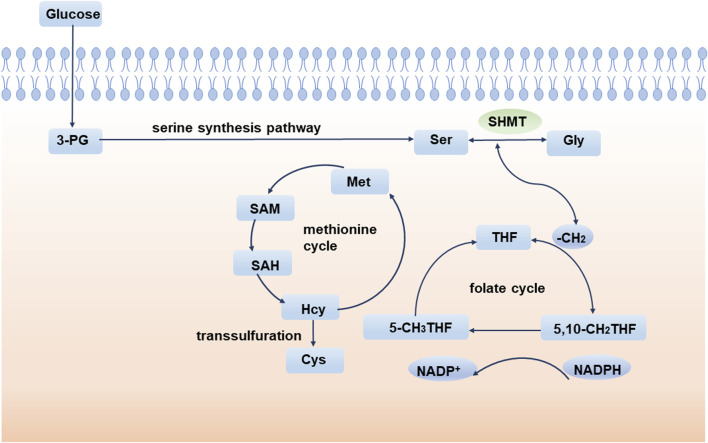
SSP and one-carbon metabolism. During the conversion of serine to glycine, a methylene group breaks off from serine and enters the folate cycle. Ultimately, this 1C unit acts as a methyl donor in the form of a SAM. 3-PG: 3-phosphoglycerate; SHMT: serine hydroxymethyl transferase; Ser: serine; Gly: glycine; THF: tetrahydrofolate; Met: methionine; SAM: S-adenosyl methionine; SAH: S-adenosyl-L-homocysteine; Hcy: homocysteine.

The 1C unit refers to an organic group containing one carbon atom, including methylene, methenyl, methyl, and formyl (Locasale, 2013; Shetty and Varshney, 2021). The 1C unit cycle carried by tetrahydrofolate is the fundamental center of cellular metabolism. The 1C unit is not only a major source of purine and pyrimidine synthesis participating in DNA and RNA synthesis but also sustains GSH and contributes to cellular redox homeostasis, which is critical for maintaining rapid tumor cell proliferation (Ducker and Rabinowitz, 2017). Isotope labeling experiments implicate amino acids, especially serine, as major one-carbon sources (Davis et al., 2004; Newman and Maddocks, 2017). During the conversion of serine to glycine, a methylene group breaks off from serine and enters the folate cycle. With the conversion of the 1C unit carried on tetrahydrofolate (THF), the methylene group from serine enters the methionine cycle. Ultimately, this 1C unit acts as a methyl donor in the form of a SAM (Figure 2).

Homocysteine (Hcy) is an important component of the methionine cycle and is a precursor of cysteine (Cys) biosynthesis. Glutathione (GSH) is an important low molecular antioxidant in cells, which is composed of glutamic acid, cysteine, and glycine (Forman et al., 2009). Studies have shown that cysteine synthesis mediated by transsulfuration is very important to promote tumor cell growth, especially when extracellular cysteine uptake is limited (Zhu et al., 2019). GSH is one of the most abundant metabolites in cells and can maintain the redox balance of cells by scavenging and reducing reactive oxygen species and maintaining an appropriate NADPH/NADP + ratio (Forman et al., 2009; Fan et al., 2014). GSH has been implicated in aging and various human diseases, such as Alzheimer’s disease (Mandal et al., 2015; Mahajan et al., 2020), Parkinson’s disease (Li et al., 1997), and diabetes (Sekhar et al., 2011). Since GSH affects the oxidation, differentiation, proliferation, and apoptosis of cells, abnormalities in GSH are also closely related to the occurrence and development of various cancers (Traverso et al., 2013). In conclusion, abnormal 1C metabolism and intracellular redox abnormalities are important mechanisms by which serine metabolism affects tumor progression.

### Key enzymes in SSP in lung cancer

**FIGURE 3:**
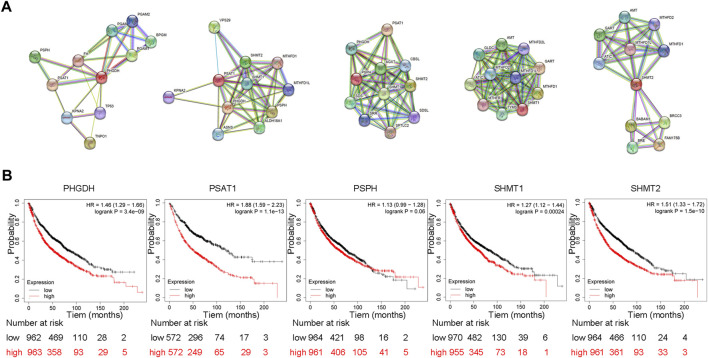
Protein interactions of SSP core enzymes and their effect on patient’s overall survival **(A)** Protein interactions of SSP core enzymes. We retrieved PHGDH, PSAT1, PSPH, SHMT1 and SHMT2 on STRING (https://cn.string-db.org/) and choose *Homo sapiens* to get the protein interaction network of SSP key enzymes in human cells. **(B)** SSP core enzyme’s function influences patient survival rates in lung cancer. The prognostic value of PHGDH, PSAT1, PSPH, SHMT1 and SHMT2 expression was analyzed using Kaplan-Meier Plotter (http://kmplot.com/analysis/). The results showed that the overall survival of patients with high expression of PHGDH, PSAT1, SHMT1 and SHMT2 was significantly decreased.

The morbidity and mortality rates of lung cancer are the highest among all malignant tumors. Recent research suggests that numerous metabolic abnormalities are involved in the development of lung cancer (Ma et al., 2021; Morrissey et al., 2021). Abnormal metabolism promotes the progression of lung cancer, and this dependence on abnormal metabolism also provides the biochemical basis for the specific killing of lung cancer. Compared with other types of cancer, lung cancer has more targeted therapy and immunotherapy drugs, and its treatment plan is more complex. However, lung cancer cells are often resistant to conventional antitumor therapies because the metabolic heterogeneity of lung cancer leads to metabolic symbiosis and therefore causes poor treatment outcomes (Yoshida, 2015). Abnormal serine metabolism, particularly the enhancement of SSP, is prevalent in lung cancer (Chen et al., 2019). Elevation of key SSP enzymes, such as PHGDH, PSAT1, and PSPH, is an important factor in the malignant progression of lung cancer cells and cancer drug resistance. In addition, these key enzymes can interact with other signaling pathways to promote lung cancer progression (Figure 3). Thus, the study of abnormal serine metabolism in lung cancer is a new way to solve the problems of lung cancer-targeted therapy resistance and immunotherapy tolerance.

### PHGDH

PHGDH is the rate-limiting enzyme in the first step of SSP and catalyzes the conversion of 3-PG, an intermediate in glycolysis and gluconeogenesis, to 3-phosphohydroxypyruvate (3-PP). NAD is an important cofactor for this process. PHGDH is highly expressed in various tumors and is associated with tumorigenesis, drug resistance, and a poor prognosis (Possemato et al., 2011; Ma et al., 2021) (Table 1). For example, PHGDH is upregulated in platin-resistant ovarian cancer and is regulated by the RNA-binding protein DDX3X and lncRNA RMRP (Bi et al., 2021). PHGDH is an indispensable factor in breast cancer pulmonary metastasis, which elevates the mTOR complex 1 (mTORC1) signaling pathway and defines sensitivity to rapamycin in tumor metastases (Rinaldi et al., 2021). Meanwhile, PHGDH is also a critical molecule of hepatocellular carcinoma resistance to sorafenib. High expression of PHGDH inhibits sorafenib-induced apoptosis of liver cancer cells by activating SSP and inhibiting alpha-ketoglutarate (α-KG), serine, and NADPH (Wei et al., 2019).

**TABLE 1:** Function and potential mechanism of PHGDH in different tumors.

Cancer	Function	Mechanism	Ref
acute myeloid leukemias	affect the sensitivity of FLT3-ITD acute myeloid leukemias to cytarabine	stimulate serine biosynthesis	Bjelosevic et al. (2021)
breast cancer	increase cell proliferation in cell lines with elevated PHGDH expression	promote serine pathway flux and anaplerosis of glutamate into the TCA cycle	Possemato et al. (2011)
breast cancer	affect pulmonary metastasis and sensitivity to rapamycin	elevate mTORC1 signaling pathway	Rinaldi et al. (2021)
breast cancer	PHDGH heterogeneity potentiates metastatic dissemination	activate the hexosamine–sialic acid pathway	Rossi et al. (2022)
colorectal cancer	enhance the antitumor activity of 5-FU	inhibit 5-FU-induced cell death, DNA damage, and metabolic perturbations	Montrose et al. (2021)
hepatocellular carcinoma	affect sorafenib resistance	activate SSP and inhibit α-KG, serine, and NADPH	Wei et al. (2019)
lung adenocarcinoma	facilitate erlotinib resistance	promotes GSH metabolism through the SSP pathway	Dong et al. (2018)
pancreatic cancer	contribute to cell proliferation, migration and invasion	up-regulate cyclin B1, cyclin D1, MMP-2, and MMP-9	Song et al. (2018)
pancreatic cancer	promote cell proliferation and tumorigenesis	enhance the translation initiations by interacting with eIF4A1 and eIF4E	Ma et al. (2019)
pancreatic ductal adenocarcinoma	promote tumor growth	enhance serine biosynthesis	Itoyama et al. (2021)

PHGDH is also a high-expression proto-oncogene in lung cancer that promotes cancer progression by activating serine synthesis. Proteomic analysis of six small cell lung carcinoma (SCLC) and six pulmonary carcinoid tumor (PCT) tissues indicated that PHGDH overexpression is significantly associated with cancer metabolism and poor overall survival (OS) (Fujii et al., 2018). Quantitative reverse transcriptase polymerase chain reaction (qRT-PCR) and immunohistochemistry experiments in 319 NSCLC samples and 143 control samples further showed that high expression of PHGDH was significantly correlated with pathological features of patients, such as lymph node metastasis (*p* = .021) and TNM stage (*p* = .016) (Zhu et al., 2016).

Bioinformatics analysis of 720 lung adenocarcinoma patients and tissue microarray analysis of 75 lung adenocarcinoma (LUAD) and adjacent normal tissues revealed that PHGDH is a metabolic subtype of LUAD. 13C isotopomer flux analysis demonstrated that these cells maintain a higher level of glycolysis and generate more serine from glucose. Compared with low-PHGDH-expressing cells, high-PHGDH-expressing cells maintain the characteristics of rapid proliferation and migration. This is mainly because enhanced serine metabolism promotes purine and pyrimidine precursor synthesis for massive DNA synthesis in rapidly proliferating tumor cells (Zhang et al., 2017). Moreover, the expression of PHGDH is required for the erlotinib-resistant LUAD cell lines PC9ER4 and HCC827ER9 (Dong et al., 2018). Inhibition of PHGDH re-sensitizes LUAD cells resistant to erlotinib treatment. Bioinformatic analysis and experiments showed that overexpression of PHGDH promoted GSH metabolism through the SSP pathway and downstream methionine cycle. GSH counteracts reactive oxygen species (ROS) and further inhibits the damage caused by chemotherapeutic drugs to the DNA, proteins and lipids of tumor cells.

Regulation of epigenetic pathways is an essential mode of regulation of PHGDH. Parkin, an E3 ubiquitin ligase, ubiquitinates PHGDH at lysine 330, causing PHGDH degradation and attenuating serine synthesis. Consequently, low expression of Parkin in lung cancer contributes to the high expression of PHGDH (Liu et al., 2020). In colorectal cancer, PHGDH is also monoubiquitinated by cullin 4A–based (Cul4A-based) E3 ligase complex at lysine 146. Conversely, by recruiting DnaJ homolog subfamily A member 1 (DNAJA1), K146 monoubiquitination (K146mUb) enhances PHGDH activity rather than promotes PHGDH degradation (Zhang et al., 2021a).

Post-transcriptional regulation by non-coding RNAs also plays a pivotal role in modulating PHGDH expression. Studies have shown that the circ_0062682/miR-940/PHGDH axis promotes serine metabolism and tumorigenesis in colorectal cancer, which may be a potential novel therapeutic target (Sun et al., 2021).

### PSAT1

PSAT1 is the second key enzyme in SSP and catalyzes the conversion of 3-PP to 3-phosphoserine (3-PS). The essential transamination reaction in which glutamate is converted to α-KG occurs simultaneously. PSAT1 is generally overexpressed in malignant tumors (Feng et al., 2022), and plays an important role in regulating tumor progression (Table 2). For instance, PSAT1 is highly expressed in ovarian cancer and is a candidate subtype-specific biomarker suggesting that the tumor is most likely a clear cell carcinoma (Toyama et al., 2012; Zheng et al., 2019a). PSAT1 is also the top-ranked upregulated gene in colorectal cancer (CRC), which is relevant to poor chemotherapy response and prognosis of patients (Qian et al., 2017). Cell cycle analysis suggested that PSAT1 modulates chemotherapy sensitivity by inhibiting cell death and promoting recovery from oxaliplatin-induced G2/M arrest (Zhang et al., 2020).

**TABLE 2:** Function and potential mechanism of PSAT1 in different tumors.

Cancer	Function	Mechanism	Ref
breast cancer	promote cell cycle progression	up-regulate cyclin D1 *via* the GSK3β/β-catenin pathway	Gao et al. (2017)
ER-negative breast cancer	promote distant metastasis	activate Notch and β-catenin signaling pathways	Zhu et al. (2022)
triple-negative breast cancer	promote cell motility and migration	alter the F-actin cytoskeletal arrangement and morphology	Metcalf et al. (2020)
colorectal cancer	modulate chemotherapy sensitivity	promote the recovery from oxaliplatin-induced G2/M arrest	(Vié et al., 2008; Qian et al., 2017; Zhang et al., 2020)
epithelial ovarian cancer	inhibit oxidative stress, and promote tumor growth	increase GSH and NADPH flux and inhibit oxidative stress	Zhang et al. (2020)
esophageal squamous cell carcinoma	promote cell proliferation and invasion	upregulate the expression and activity of GSK3β/Snail	Liu et al. (2016)
non-small cell lung cancer	promote cell proliferation and tumorigenesis	inhibit cyclin D1	Yang et al. (2015)
lung adenocarcinoma	inhibit metastasis	inhibit the IFNγ/STAT1/IRF1/IFIH1-axis	Chan et al. (2020)

PSAT1 is highly expressed in NSCLC and promotes cell cycle progression, cell proliferation, and tumorigenesis. Cyclin D1 is a type of D-type cyclin that regulates the cell cycle in G1 phase and G1-S phase, thereby controlling cell proliferation (Tchakarska and Sola, 2020). Western blotting (WB) experiments suggest that knockdown of PSAT1 inhibits cyclin D1 expression and decreases Rb phosphorylation and early 2 factor (E2F) transcription activity. PSAT1 inhibits the activity of serine/threonine protein kinase glycogen synthase kinase 3b (GSK-3B) by promoting its phosphorylation at Ser-9. Phosphorylation of GSK-3B can inhibit the degradation of cyclin D1 by inhibiting its phosphorylation at Thr-286. Therefore, the overexpression of PSAT1 promotes the Cyclin D1 activity but does not affect cyclin D1 expression at the mRNA level (Yang et al., 2015). In addition, PSAT1 promotes the nuclear translocation of pyruvate kinase M2 (PKM2) in response to EGFR activation, thus promoting lung cancer progression (Biyik-Sit et al., 2021).

Furthermore, PSAT1 is highly expressed in LUAD and is correlated with clinicopathological events and poor clinical outcomes. By analyzing PSAT1-based transcriptomics microarray chips, it was found that interferon regulatory factor 1 (IRF1) and its downstream protein, interferon induced with helicase C domain 1 (IFIH1), are inhibited by the overexpression of PSAT1. The results of Ingenuity Pathway Analysis (IPA) and relative luciferase activity further suggest that when PSAT1 is overexpressed, the activities of interferon-γ (IFNγ) and signal transducer and activator of transcription 1 (STAT1), the upstream factors of IRF1, are inhibited. Further studies have found that overexpression of PSAT1, inhibits the phosphorylation of STAT1 at Y701 and S727, resulting in the suppression of dimerization and DNA binding of STAT1. In conclusion, PSAT1 inhibits LUAD metastasis by inhibiting the IFNγ/STAT1/IRF1/IFIH1-axis and leads to poor prognosis in LUAD patients (Chan et al., 2020). Furthermore, in patients with EGFR inhibitor resistance, the abnormal upregulation of PSAT1 inhibits the ROS-dependent JNK/c-Jun pathway, thereby inhibiting cell apoptosis. PSAT1 interacts with IQGAP1 and stimulates STAT3-mediated cell migration. In general, high expression of PSAT1 not only promotes tumor metastasis, but also causes resistance to EGFR inhibitors, which together leads to poor prognosis in LUAD patients (Luo et al., 2022).

The expression of PSAT1 is also regulated by multiple mechanisms. As mentioned earlier, ATF4 is a canonical regulator that promotes PSAT1 overexpression (Gao et al., 2017). In addition, the transcriptional regulators TAZ and YAP (TAZ/YAP) induce PSAT1 expression to drive oncogenic traits, such as metastasis and drug resistance (Mohamed et al., 2016; Yang et al., 2018a; Kim et al., 2022). Moreover, the luciferase assay suggested that miRNAs miR-145–5p (Ding et al., 2022), miR-340 (Yan et al., 2015), and miR-424 (Fang et al., 2018) inhibit the expression of PSAT1 by directly targeting the 3′untranslated regions. There are also studies indicating that long non-coding RNA RP4-694A7.2 (Fan et al., 2021), MEG3 (Li et al., 2020a) and MEG8 (Guo et al., 2021) modulate PSAT1 expression.

### PSPH

PSPH, also known as PSP or PSPHD, belongs to a subfamily of phosphotransferases. PSPH is the last rate-limiting enzyme of SSP and is responsible for the conversion of phospho-L-serine to L-serine. Recent studies have shown that PSPH is highly expressed in a variety of cancers and mediates malignant behaviors such as tumor proliferation, metastasis, and poor prognosis (Jovov et al., 2012; Parada et al., 2017) (Table 3).

**TABLE 3:** Function and potential mechanism of PSPH in different tumors.

Cancer	Function	Mechanism	Ref
cutaneous squamous cell carcinoma	promote SCC cell proliferation	increase cyclin D1 levels	Bachelor et al. (2011)
colorectal cancer	promote metastasis and inhibit sensitivity to 5-FU treatment	promote serine conversion to GSH and inhibit 5-FU-induced ROS	(Li et al., 2016; Sato et al., 2017)
endometrial carcinogenesis	regulate tumor progression	synthesize important metabolic intermediates	Strunck et al. (2001)
gastric cancer	lead to tumor low immune score	affect the density of immune cells	Huang et al. (2022)
hepatocellular carcinoma	induce cell autophagy and promote cell proliferation and invasion	regulate AMPK/mTOR/ULK1 signaling pathway	Zhang et al. (2021b)
melanoma	promote melanoma growth and metastasis	inhibit 2-HG, thus increasing 5 hmC and reducing H3K4me3 modifications	Rawat et al. (2021)
non-small cell lung cancer	mediate the metastasis and proliferation	promote MAPK signaling pathway	Liao et al. (2019)
T-cell acute lymphoblastic leukemia	promote proliferation of T-ALL cell lines and their capacity to expand	drive the induction of serine biosynthesis	Kampen et al. (2019)

PSPH is also an upregulated oncogene in NSCLC that regulates tumor progression and correlates with the clinical stage and pathological features. Using qRT-PCR experiments in 73 pairs of NSCLC and adjacent non-tumorous tissues, it was found that PSPH expression level is associated with TNM stage (*p* < .01) and lymph node and/or distal metastasis (*p* < .05). Cell function experiments suggested that PSPH enhance NSCLC cell proliferation and migration and promote the cell cycle in the G2-M phase. WB experiments showed that after inhibiting PSPH expression, phosphorylation rather than total protein levels of ERK, MEK, and P38 is suppressed, indicating that PSPH promotes NSCLC metastasis through the MAPK signaling pathway (Liao et al., 2019).

Insulin receptor substrate 1 (IRS1) is a protein phosphorylated by insulin receptor tyrosine kinase, which acts as an essential regulator in the progression of metabolic diseases (Copps and White, 2012). Moreover, IRS1 regulates the development of drug resistance in various tumors, such as breast cancer (Choi et al., 2019), and has attracted widespread attention as a target for tumor-targeted therapy (Hao et al., 2014). Proteomic analyses indicated that IRS1 might be a specific substrate of PSPH. Furthermore, immunoprecipitation and immunoblot assays validated that PSPH regulates IRS1 dephosphorylation at Ser-794, and that the D20 of PSPH is the active site. The Ser-794 site of IRS1 is an inhibitor of the downstream molecules Akt and the p70 S6 kinase phosphorylation site. Therefore, overexpression of PSPH promotes activation of the PI3K/Akt/mTOR signaling pathway by inhibiting the phosphorylation of IRS1. This is an important mechanism by which PSPH promotes lung cancer progression *in vitro* and *in vivo* (Park et al., 2019).

### SHMT

SHMT sustains cell growth and proliferation in normal and tumor tissues by regulating one-carbon metabolism (Guiducci et al., 2019). In human cells, SHMT has two isoforms: SHMT1 in the cytosol and SHMT2 in the mitochondria (Tramonti et al., 2018). In the cytosol and mitochondria, SHMT1 and SHMT2 catalyze the conversion of serine and THF to glycine and 5,10-methylene tetrahydrofolates (5, 10-CH2-THF), respectively. In general, the 1C unit required for the proliferation of various cancer cells is produced by serine metabolism in the mitochondria and transported by folate. During 1C unit generation, SHMT2 directly catalyzes the conversion of serine to glycine and directs the folate cycle production. SHMT1 is likely to regulate the folate cycle and one-carbon metabolism (Giardina et al., 2018). However, studies have shown that cells with low expression of the reduced folate carrier (RFC) solute carrier family 19 member 1 (SLC19A1) are more dependent on the cytoplasmic folate cycle regulated by SHMT1 (Lee et al., 2021). This result supports the view that mitochondrial folic acid metabolism is not the sole contributor to 1C units in tumors. In summary, both SHMT1 and SHMT2 play important roles in the development of various tumors (Tables 4, 5).

**TABLE 4:** Function and potential mechanism of SHMT1 in different tumors.

Cancer	Function	Mechanism	Ref
acute lymphocytic leukemia	increase the risk of cancer	cause uracil misincorporation	Skibola et al. (2002)
colorectal cancer	increase the risk of cancer	modify thymidylate synthesis capacity	Macfarlane et al. (2011)
lung cancer	induce apoptosis in lung cancer cells	cause uracil misincorporation	(Pandey et al., 2014; Paone et al., 2014)
ovarian cancer	promote tumor growth and cell migration	control the expression of pro-oncogenic inflammatory cytokines by regulating sialic acid Neu5Ac	Gupta et al. (2017)

**TABLE 5:** Function and potential mechanism of SHMT2 in different tumors.

Cancer	Function	Mechanism	Ref
bladder cancer	support cell growth, regulate cell cycle and apoptosis	promote the accumulation of intracellular ROS, activate caspase-3, regulate STAT3 signaling	(Zhang and Yang, 2021; Zhang et al., 2022a)
breast cancer	enhance breast cancer resistance to lapatinib	promote mitochondrial metabolic adaption	Li et al. (2020b)
colorectal cancer	promote CRC cell proliferation, invasion and migration	interact with β-catenin in the cytoplasm and inhibit the ubiquitylation-mediated degradation of β-catenin	Liu et al. (2021)
colorectal cancer	regulate 5-FU chemoresistance in CRC	prevent cytosolic p53 degradation by inhibiting the binding of p53 and HDM2	Chen et al. (2021)
kidney cancer	cause poor prognosis in kidney cancer	increase the expression of NDUFA4L2	Wang et al. (2020)
lung cancer	enhance tumor proliferation	regulate 1C metabolism	Yang et al. (2018b)
lymphoma	amplification of SHMT2 in cooperation with BCL2 initiates lymphomagenesis	catalyze the conversion of serine to glycine and produce an activated 1C unit	Parsa et al. (2020)
Burkitt lymphoma	regulate tonic BCR signaling	stabilize the TCF3 transcriptional survival program	Wilke et al. (2022)
oral squamous cell carcinoma	promote cell viability, migration, invasion	bind to ILF2	Zhang et al. (2022b)

In lung cancer, high SHMT1 expression promotes cell proliferation. SHMT1 knockdown causes cell cycle arrest and p53-dependent apoptosis. Further experiments have indicated that reducing the expression of SHMT1 can reduce dTMP synthesis, leading to increased abnormal uracil accumulation during DNA replication. This causes high genomic instability and DNA strand breaks. More importantly, this type of apoptosis cannot be rescued by adding glycine or serine to the medium. These results suggest that targeting SHMT1 may contribute to the treatment of patients with lung cancer (Pandey et al., 2014; Paone et al., 2014).

SHMT2 is a proto-oncogene that is highly expressed in various human carcinogenesis (Lee et al., 2014), such as breast cancer (Bernhardt et al., 2017), kidney renal papillary cell carcinoma, liver hepatocellular carcinoma, and gastrointestinal tumors (Liu et al., 2019). Kyoto Encyclopedia of Genes and Genomes (KEGG) pathway analysis indicates that SHMT2 is enriched in 5 pathways including one carbon pool by folate (hsa00670), metabolic pathways (hsa01100), biosynthesis of antibiotics (hsa01130), glyoxylate and dicarboxylate metabolism (hsa00630), and glycine, serine, and threonine metabolism (hsa00260). Moreover, the expression of SHMT2 is related to the clinicopathological characteristics of the tumor, such as the patient’s age and tumor stages (Usman et al., 2022).

In lung cancer, SHMT2 is also a highly expressed oncogene, and its expression is highly related to tumor-infiltrating lymphoytes (Luo et al., 2021) and shorter OS (Koseki et al., 2018). SHMT2 maintains lung cancer development mainly through classical regulation of 1C metabolism. Other specific mechanisms have not yet been identified. SIRT5, a sirtuin, binds to SHMT2 and mediates its desuccinylation at lysine 280. Since hypersuccinylation of SHMT2 inhibits its enzymatic activity, SIRT5 can enhance the promotion of SHMT2 on tumor proliferation (Yang et al., 2018b).

### Targeting SSP in the treatment of tumor

Many tumors show serine dependence and serine starvation triggers serine synthesis from glucose and glycine, which causes altered metabolism compared to that in normal tissues (Li and Ye, 2020; Tajan et al., 2021). Serine/glycine uptake is thought to be closely related to cancer cell proliferation, as serine and glycine are interconverted under the catalysis of SHMT. Further experiments have shown that the uptake of serine, rather than glycine, supported the 1C unit metabolism. The uptake of glycine cannot substitute serine to promote cell proliferation alone (Labuschagne et al., 2014). Studies have shown that both serine deprivation and excess glycine inhibit cell proliferation (Li et al., 2021a). This phenomenon is related to the production of 5,10-methylene-tetrahydrofolate, which is supported during the conversion of serine to glycine and is depleted when glycine is converted to serine (Maddocks et al., 2013). However, high expression of PHGDH, PSAT1, and PSPH is ubiquitous in tumor cells, which activates endogenous serine metabolism and thus weakens the effect of serine starvation on tumor treatment. PHGDH is the first key enzyme of SSP that plays a cancer-promoting role in multiple tumors. Experiments show that a combination of PHGDH inhibitor (PHGDHi) and medium lacking serine and glycine (-SG) impedes tumor growth by inhibiting DNA, purine and GSH synthesis (Montrose et al., 2021). More importantly, supplementation with 1C unit or glycine alone did not rescue tumor cell proliferation. Adding glycine and 1C units simultaneously can partially rescue cell proliferation by recovering ATP and GTP synthesis. Furthermore, PHGDHi/-SG treatment reduced global protein synthesis, the protective response to serine depletion *in vitro* and showed better therapeutic results than monotherapy *in vivo* (Tajan et al., 2021).

Even if exogenous serine is sufficient, inhibiting the expression of key SSP enzymes has anti-tumor effects on some specific cancers, which provides new targets for tumor precision therapy (Pacold et al., 2016). The currently studied drugs that inhibit key SSP enzymes are summarized in Table 6. For instance, NCT-503 (IC50 = 2.5 ± .6 μM) is synthesized and identified as a potent PHGDH inhibitor that inhibits intracellular serine and glycine in stably overexpressing PHGDH MDA-MB-231 cells (MDA-MB-231-PHGDH). NCT-503 does not regulate other amino acids such as aspartate. Similarly, isotope labeling experiments suggest that NCT-503 only controls SSP and 1C unit metabolism, but does not affect the process of glycolysis to 3-PG. Cellular experiments indicated that knockout of PHGDH by NCT-503 is selectively toxic to PHGDH-dependent cell lines, but not to other PHGDH-independent cell lines. Therefore, NCT-503 may be used as a targeted therapy for tumors, such as lung cancer, breast cancer, and other tumors that highly express PHGDH (Pacold et al., 2016). Notably, most drugs identified as SSP inhibitors have not yet been used in clinical treatments. Studies on drug reuse have also identified some clinical drugs that inhibit SSP, but this pharmacological effect has not been incorporated into clinical treatment.

**TABLE 6:** Drugs targeting SSP core enzymes.

Drug	Target	Research stage	Ref
A-485, B029-2	P300	Preclinical	Cai et al. (2021)
CBR-5884	PHGDH	Preclinical	Mullarky et al. (2016)
NCT-502, NCT-503	PHGDH	Preclinical	Pacold et al. (2016)
BI-4924	PHGDH	Preclinical	Weinstabl et al. (2019)
Azacoccone E	PHGDH	Preclinical	Guo et al. (2019)
PH-739-003N, PH-755-003 N	PHGDH	Preclinical	Rodriguez et al. (2019)
WQ-2101	PHGDH	Preclinical	Wang et al. (2017)
Ixocarpalactone A	PHGDH	Preclinical	Zheng et al. (2019b)
oridonin	PHGDH	Preclinical	Tan et al. (2021)
withangulatin A	PHGDH	Preclinical	Chen et al. (2022)
thimerosal	PHGDH	Preclinical	Geeraerts et al. (2021)
regorafenib	PSAT1	Preclinical	Jiang et al. (2020)
SHIN1	SHMT	Preclinical	Ducker et al. (2017)
SHIN2	SHMT	Preclinical	García-Cañaveras et al. (2021)
sertraline	SHMT	Preclinical	Geeraerts et al. (2021)
RZ-2994	SHMT1, SHMT2	Preclinical	Pikman et al. (2022)
lometrexol, pemetrexed	SHMT2	Preclinical	Scaletti et al. (2019)
5-substituted pyrrolo [2,3-day] pyrimidines AGF291, AGF320 and AGF347	SHMT2	Preclinical	Dekhne et al. (2019)
metformin	SHMT2	Preclinical	Tramonti et al. (2021)

Moreover, the combination of SSP key enzyme inhibitors and chemotherapy drugs will achieve better therapeutic effects (Jing et al., 2015; Ross et al., 2017; Li et al., 2021b). For example, in bladder cancer, compared to individual agents, PHGDH inhibition promotes gemcitabine/cisplatin-induced antitumor effects by suppressing serine biosynthesis and inhibiting cancer cell viability (Yoshino et al., 2020).

Drugs can also regulate the SSP pathway by affecting the post-translational modification of key enzymes, thereby exerting tumor suppressor effects. P300/CBP is a crucial epigenetic regulator of glycolysis and SSP metabolic enzymes that acetylate histone H3K18/K27 during transcriptional activation. A-485 and B029-2 are two selective and highly potent p300 inhibitors that suppress tumor cell metabolism and tumor progression by targeting p300/CBP and reducing the levels of key metabolic enzyme genes, such as PSPH, PSAT1 promoter region H3K18Ac, and H3K27Ac. Therefore, A-485 and B029-2 could be used as potential therapeutic strategies for lung cancer, liver cancer, and other metabolically abnormal tumors (Cai et al., 2021).

Remarkably, most drugs targeting key SSP enzymes inhibit tumor cell proliferation by inhibiting endogenous serine metabolism, but there are also some special mechanisms (Zhao et al., 2020; Arlt et al., 2021). For example, the multi-kinase inhibitor regorafenib directly stabilizes PSAT1 to maintain its high expression, thereby activating PRKAA-dependent autophagy. Therefore, high PSAT1 expression is essential for regorafenib to kill tumor cells (Jiang et al., 2020).

## Conclusion

Studies have shown that even if exogenous serine is sufficient, *de novo* serine biosynthesis is required in tumor cells (Reid et al., 2018). Serine is an important node in cancer cell metabolism (Mattaini et al., 2016). SSP provides serine to cancer cells for protein synthesis. SSP, together with glycolysis and 1C metabolism, forms a metabolic network that is crucial for the occurrence of tumors (Reina-Campos et al., 2020). Specifically, SSP is an important destination for the glycolytic intermediate 3-PG. After 3-PG enters SSP, it no longer provides energy for cancer cells but is converted into serine through a three-step catalytic reaction, and finally provides a carbon source for 1C metabolism. Thus, cellular glucose participates in molecular synthesis rather than providing energy. Although cell replication requires energy, to maintain a high level of 3-PG to support anabolic reactions in cells, aerobic glycolysis is also important for cancer cells with rapid proliferation (Li and Zhang, 2016). Consequently, this metabolic mode partly explains the Warburg effect in tumor cells. Notably, although *de novo* serine synthesis may provide a type of flow for glycolytic intermediates, this requirement for serine synthesis is not the only reason for the unusual uptake of glucose in tumors.

PHGDH is the first rate-limiting enzyme of SSP and largely determines its flow. The key SSP enzymes PHGDH, PSAT1, and PSPH are significantly overexpressed in a variety of cancers and are associated with poor patient outcomes. In the past, mechanistic research and drug development have mainly targeted PHGDH. No known metabolic reaction can bypass PSAT1 or PSPH to complete the downstream reaction catalyzed by PHGDH (Buqué et al., 2021). Drugs targeting PSAT1 and PSPH may function similarly to those targeting PHGDH. Here, we propose that the use of targeted drugs according to the different expression levels of key SSP enzymes in different tumors is the next research direction.

The study of tumor metabolism originated with the discovery of the Warburg effect (Warburg et al., 1927; Warburg, 1956). With the discovery of numerous oncogenes, cancer is recognized as a genetic disease (Bignell et al., 2010). The discovery of oncogenes has greatly expanded tumor treatment and improved patient survival. However, the mortality rate of cancer is still high, especially for patients who fail to be diagnosed early (Miller et al., 2022; Siegel et al., 2022). In recent years, owing to the application of metabolomics and the discovery of “oncometabolites,” cancer, as a kind of metabolic disorder, has attracted the attention of researchers. This is of great significance in the diagnosis and treatment of cancer. For example, the detection of metabolic abnormalities can indicate early cancer development. Drugs targeting key enzymes involved in abnormal metabolism can also inhibit the progression of some tumors (Wishart, 2015).

Metabolomics studies enable the simultaneous detection of many small-molecule metabolites, thus helping to assess metabolic abnormalities in lung cancer (Noreldeen et al., 2020). It is now clear that there are many metabolic abnormalities in lung cancer, such as abnormal energy metabolism (Vanhove et al., 2019), dysregulation of lipid metabolism (Wang et al., 2022), and glucose metabolism disorder (Ding et al., 2019; Smolle et al., 2020). Current research focuses on the occurrence and development mechanism of abnormal metabolism in lung cancer, its impact on tumor progression, and its clinical application. It is of great significance to promote metabolomic measurement data to provide evidence for early diagnosis and pathological classification of lung cancer. According to the different types and stages of lung cancers with different metabolic abnormalities, research on new targeted drugs and reducing drug resistance during lung cancer treatment has very broad prospects for clinical application.
